# Chronic Granulomatous Disease: a Cohort of 173 Patients—10-Years Single Center Experience from Egypt

**DOI:** 10.1007/s10875-023-01541-4

**Published:** 2023-07-11

**Authors:** Dalia Abd Elaziz, Rabab EL Hawary, Safa Meshaal, Radwa Alkady, Sohilla Lotfy, Alia Eldash, Aya Erfan, Engy Chohayeb, Mai Saad, Jeannette Boutros, Nermeen Galal, Aisha Elmarsafy

**Affiliations:** 1 Pediatric Department, Faculty of Medicine, Cairo University, Cairo, Egypt; 2 Clinical Pathology Department, Faculty of Medicine, Cairo University, Cairo, Egypt

**Keywords:** X-linked CGD, AR-CGD, Autoimmune manifestations in CGD, Infections in CGD, CGD in Egypt

## Abstract

**Purpose:**

Chronic granulomatous disease (CGD) is an inherited primary immunodeficiency disorder of phagocytes, characterized by recurrent fungal and bacterial infections. Our aim is to describe the different clinical presentations, non-infectious auto-inflammatory features, types and sites of infections, and to estimate the mortality among our large cohort.

**Methods:**

This is a retrospective study conducted at the Pediatric Department of Cairo University Children’s Hospital in Egypt, including cases with a confirmed CGD diagnosis.

**Results:**

One hundred seventy-three confirmed CGD patients were included. AR-CGD was diagnosed in 132 patients (76.3%) including 83 patients (48%) with p47^phox^ defect, 44 patients (25.4%) with p22^phox^ defect, and 5 patients (2.9%) with p67^phox^ defect. XL-CGD was diagnosed in 25 patients (14.4%). The most common recorded clinical manifestations were deep-seated abscesses and pneumonia. Gram-negative bacteria and *Aspergillus* were the most frequently isolated species. Regarding the outcome, 36 patients (20.8%) were lost from follow-up. Among patients with known outcome, 94/137 patients (68.6%) are living, while 43/137 patients (31.4%) died.

**Conclusion:**

AR-CGD is predominant in Egypt; CGD must always be ruled out in any patient presenting with typical or atypical mycobacterial or BCG-disease.

**Supplementary Information:**

The online version contains supplementary material available at 10.1007/s10875-023-01541-4.

## Introduction

Chronic granulomatous disease (CGD) is an inherited primary immunodeficiency disorder (PID) of phagocytes, characterized by a defect of nicotinamide adenine dinucleotide phosphate (NADPH) oxidase components [1, 2]. These are responsible for the production of superoxide anion and hydrogen peroxide through the transfer of electrons from NADPH in the cytosol across the phagosome membrane and the delivery of protons that produce hydrogen peroxide. The produced reactive oxygen intermediates (ROI) damage the phagocytosed microorganisms [3]. The enzyme is formed of five subunits: gp*91*^phox^ (*CYBB*) and p*22*^phox^ (*CYBA*), which are integral membrane proteins that form the flavocytochrome b588 (the electron transport center of the enzyme [4] and p47^phox^ neutrophil cytosolic factor 1(*NCF1*), p67^phox^ neutrophil cytosolic factor 2(*NCF2*), p40^phox^ neutrophil cytosolic factor4 (*NCF4*) which are cytosolic components [5, 6]. A CYBC1 gene mutation leads to reduced expression of NADPH oxidase main subunit (gp91phox) and may result in CGD [7]. The gene encoding gp91^phox^ is found on the X chromosome, while the genes of the other proteins are located on autosomes [8]. CGD is characterized by recurrent fungal and bacterial infections [9, 10] and hyper-inflammatory and autoimmune symptoms [11, 12].

Laboratory diagnosis of CGD can be made by the nitroblue tetrazolium dye reduction test (NBT) [13] or by flow-cytometry-based dihydrorhodamine (DHR) assay, which is the preferred screening test due to its higher reproducibility, sensitivity, rapidity, and ability to detect X-linked carriers [14, 15]. Few reports were published about CGD in Egypt [15, 16]. For the paucity of data on the disease burden in Egypt, we present a large cohort of patients through our 10 years of experience.

## Patients and Methods

This is a retrospective study conducted at the PID Unit, Pediatric Department of Cairo University Children’s Hospital, Egypt, from 2011 to 2021. The study included cases with a confirmed CGD diagnosis. Detailed history and clinical examination were recorded as well as the microbiological workup and the vaccination history. Glucose-6-phosphate dehydrogenase (G6PD) deficiency was excluded as none of the patients suffered from hemolytic anemia, and all the patients proved to be deficient in one of the NADPH components.

### Dihydrorhodamine Assay Technique

Patients were confirmed to be diagnosed as CGD based on dihydrorhodamine assay by flow cytometry. Mothers of male patients were tested to differentiate XL from AR-CGD if the patient showed defect in expression of both gp91^phox^ and p22^phox^. A mean fluorescence stimulation index (SI) was calculated. SI of 70 was considered the cutoff in our laboratory [15, 16].

### Intracellular Staining of Neutrophil NADPH Components

The test was done for 160 patients on whole blood samples. Monoclonal antibodies to NOXA2/p67^phox^ (ab109523), NCF1/p47^phox^ (ab179457), cytochrome b245 light chain antibody/p22^phox^ (ab87736), and NOX2/gp91^phox^ antibody (ab80508) were utilized as previously reported [15, 17].

### Molecular Analysis

Targeted genetic diagnosis using Sanger sequencing was done for 44 patients based on the results of the intracellular NADPH components assessment by flow cytometry as previously reported [18].

### Infections Profile

BCG-itis was defined as a local abscess or severe ulcer at the site of injection, and regional BCG-itis was defined as the involvement of regional lymph nodes, including enlargement, suppuration, and/or fistula formation. Disseminated BCG-osis was defined as the presence of BCG at more than one remote site and a positive blood and/or bone marrow culture [19–21].

Tuberculosis was diagnosed according to the criteria proposed by Graham et al. [22]. Mycobacterial infections were diagnosed based on clinical and radiologic findings, staining for acid-fast bacilli, supportive histology, immunologic evidence of *Mycobacterium tuberculosis*, and microbiological culture results when available.

Fungal infections were suspected and diagnosed according to the Revised Definitions of Invasive Fungal Disease from the European Organization for Research and Treatment of Cancer/Invasive Fungal Infections Cooperative Group and the National Institute of Allergy and Infectious Diseases Mycoses Study Group (EORTC/MSG) Consensus Group [23].

### Prenatal Diagnosis and Genetic Counseling

Genetic counseling was offered for families, and prenatal diagnosis was done in 4 families with a history of one or more children affected with CGD [24, 25].

### Statistical Methods

Data were analyzed using Statistical Package for the Social Science, release 15 (SPSS Inc., Chicago, IL), and *p*-values < 0.05 were considered statistically significant.

## Results

One hundred seventy-three patients from 149 different kindreds were diagnosed with CGD based on DHR test results: 107 males (61.8%) and 66 females (38.2 %). Based on the defective NADPH component, autosomal recessive CGD (AR-CGD) was diagnosed in 132 patients (76.3%) (70 males and 62 females). This included 83 patients (48%) with p47^phox^ defect, 44 patients (25.4%) with p22^phox^ defect, and 5 patients (2.9%) with p67^phox^ defect. X-linked CGD (XL-CGD) was diagnosed in 25 patients (14.4%); all have maternal carrier DHR pattern.

**Fig. 1:**
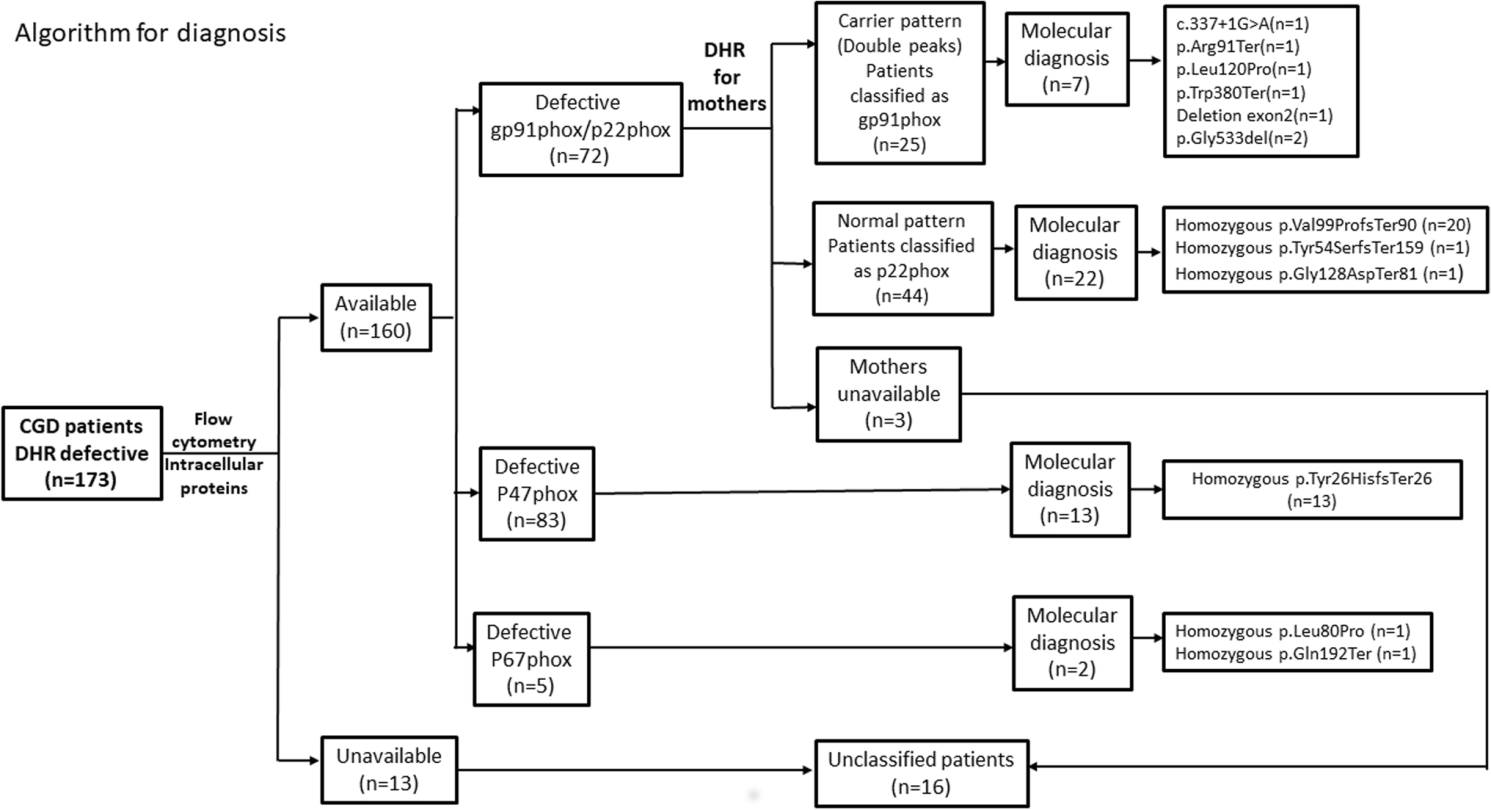
An algorithm showing the methods for patients’ diagnosis and classification

Meanwhile, 16 patients (9.2%) could not be categorized; this included 3 male patients with gp91^phox^ and p22^phox^ deficiency, whose mothers were not available to be tested for carrier pattern of DHR which could differentiate between P22^phox^ and gp91^phox^ deficiency. Another 13 patients (4 females and 9 males) who had abnormal DHR results could not be categorized due to the transient unavailability of testing either by the intracellular NADPH protein component expression by flow cytometry or the molecular analysis (Fig. 1).

The geographical distribution of CGD patients in Egypt was the following:

(i) Upper Egypt; 4 patients with gp91^phox^ defect, 25 patients with p47^phox^ defect, 11 patients with p22^phox^ defect, and 2 patients with p67^phox^ defect.
(ii) Cairo Metropolitan area; 6 patients with gp91^phox^ defect, 25 patients with p47^phox^ defect, 7 patients with p22^phox^ defect, and 1 patient with p67^phox^ defect.
(iii) North Coast cities; 9 patients with p22^phox^ defect.
(iv) Delta cities; 13 patients with gp91^phox^ defect, 20 patients with p47^phox^ defect, 6 patients with p22^phox^ defect, and 2 patients with p67^phox^ defect.
(v) Sinai; 9 patients with p47^phox^ defect.

While, we had some patients from nearby countries including 9 patients with p22^phox^ defect from Libya, 4 patients with p47^phox^ defect from Yemen. One patient with p22^phox^ defect, and another with XL-CGD from Palestine, one patient with p47^phox^ defect and one XL-CGD patient from Sudan, and one patient with p22^phox^ defect from Syria. It was noted that p22^phox^ deficient patients were prevalent in the North Coast cities as well as in Eastern Libyan cities, while p47^phox^ deficient patients were prevalent among the Delta, Nile cities, and Sinai. All the Yemeni patients were diagnosed with p47^phox^ defect (Supplemental Figure 1).

Positive consanguinity was reported in 141 (81.5%) patients with 91.7% consanguinity among AR-CGD. Fifty-seven (32.9%) patients had a history of previous sibling death and 28% with siblings’ affection. The median age at diagnosis was 48 months (range 1–186 months), while the median age at presentation was 7 months (range 0.2–180 months). P47^phox^ deficient patients were the oldest, while p22^phox^/gp91 deficient patients were the youngest at both presentation and diagnosis (Table 6).

### Molecular Analysis

The genetic diagnosis using Sanger sequencing was done for 44 patients based on the results of intracellular assessment of NADPH components by flow cytometry.

The variants found in the CYBA gene in 22 patients from 19 different families were all in homozygous form; c.295_301delGTGCCCG;p.Val99ProfsTer90 in 20 patients (from north coast and Libya), c.160_161 Ins C;p.Tyr54SerfsTer159 in one Palestinian patient, and c.383_393delCACTGCTCGCC;p.Gly128AspfsTer81 in another patient from upper Egypt.

Regarding the NCF1 gene, the only variant detected in 13 patients from 9 different families was (c.75_76delGT;p.Tyr26HisfsTer26) in homozygous form in all of them.

Two homozygous variants detected in the NCF2 gene in 2 patients were (c.239 T > C; p.Leu80Pro and c.574C > T; p.Gln192Ter).

While the variants found in the CYBB gene in 7 male patients from 6 different families were (c.337 + 1G > A, c.271C > T;p.Arg91Ter, c.359 T > C;p.Leu120Pro, c.1139G > A;p.Trp380Ter, Deletion Exon 2, c.1598_1600delGAG;p.Gly533del. all in hemizygous form [18].

### Clinical Manifestations

**Fig. 2:**
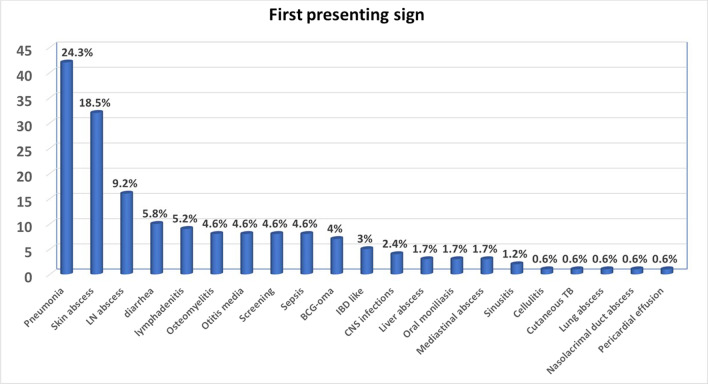
The first presenting sign in chronic granulomatous disease patients

The most common first manifestation was pneumonia, followed by skin and lymph nodes abscesses. Other presentations included diarrhea, lymphadenitis, osteomyelitis, otitis media, sepsis, regional BCG-itis, inflammatory bowel disease (IBD), CNS infection, liver abscess, oral moniliasis, mediastinal abscess, sinusitis, cellulitis, cutaneous TB, lung abscess, nasolacrimal duct abscess, and pericardial effusion. Eight patients (4.6%) were diagnosed during family screening without any symptoms (Fig. 2).

**Fig. 3:**
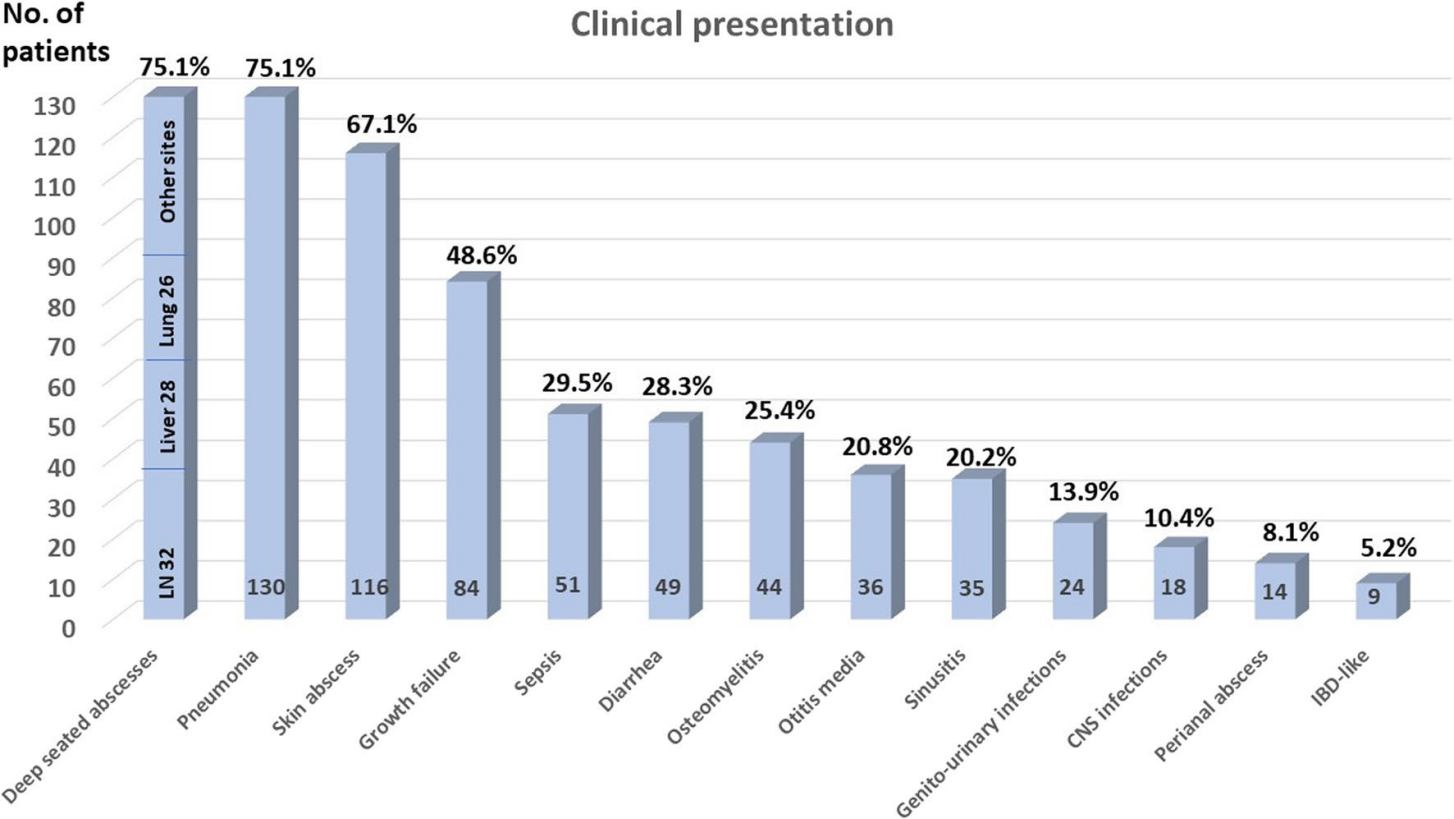
The clinical signs in chronic granulomatous disease patients

The most common recorded overall clinical manifestations were deep-seated abscesses and pneumonia, followed by growth failure, sepsis, diarrhea, osteomyelitis, otitis media, sinusitis, genito-urinary infections, CNS infections, perianal abscesses, and IBD (Fig. 3) (Table 1).

**Table 1:** The site and number of deep-seated abscesses in CGD patients

Site	gp91^phox^	p47^phox^	p22^phox^	p67^phox^	Unclassified	Total
Lymph node	3 (17.6%)	12 (19.3%)	12 (30.7%)		5 (50%)	32 (24.6%)
Liver	6 (35.3%)	12 (19.3%)	8 (20.5%)		2 (20%)	28 (21.5%)
Lung	2 (11.8%)	17 (27.4%)	7 (17.9%)			26 (20%)
Dental	1 (6%)	6 (9.6%)	2 (5.1%)			9 (6.9%)
Mediastinal	1 (6%)	6 (9.6%)	1 (2.5%)	1 (50%)		9 (6.9%)
Spleen	2 (11.8%)		4 (10.2%)		1 (10%)	7(5.3%)
Brain	1 (6%)	5 (8%)				6 (4.6%)
Joint			2 (5.1%)	1 (50%)	2 (20%)	5 (3.8%)
Empyema		1 (1.6%)	2 (5.1%)			3 (2.3%)
Pericardial		2 (3.2%)	1 (2.5%)			3 (2.3%)
Thyroid		1 (1.6%)				1 (0.8%)
Nasolacrimal duct	1 (6%)					1 (0.8%)
Total	17 (100%)	62 (100%)	39 (100%)	2 (100%)	10 (100%)	130 (100%)

Regarding the non-infectious manifestations, seventeen patients developed granulomata mostly in the liver and lung with no isolated organism from cultures, while nine patients developed IBD presentations. Four patients had non-infectious polyarticular arthritis with high inflammatory markers. Three patients developed vasculitis, one of them was drug-induced. One XL-CGD patient developed IBD at 9 months, then autoimmune hepatitis and autoimmune hemolytic anemia at 2.5 years. An 11-year-old p67^phox^ deficient patient developed autoimmune encephalitis at the age of 5 years, then developed insulin-dependent diabetes mellitus (IDDM) at the age of 7 years (Table 2).

**Table 2:** The non-infectious manifestations in CGD patients

	gp91^phox^	p47^phox^	p22^phox^	p67^phox^	Unclassified	Total
Granuloma	2 (33.3%)	12 (40%)	2 (16.7%)		1 (33.3%)	17 (30.9%)
IBD	1 (16.6%)	3 (10%)	5 (41.7%)			9 (16.4%)
Cow milk allergy			2 (16.7%)	1 (25%)	1 (33.3%)	4 (7.3%)
Arthritis		3 (10%)			1 (33.3%)	4 (7.3%)
Vasculitis		3 (10%)				3 (5.5%)
Atopic dermatitis		3 (10%)				3 (5.5%)
AIHA	1 (16.6%)		2 (16.7%)			3 (5.5%)
Uveitis-choroiditis	1 (16.6%)	1 (3.3%)				2 (3.6%)
MISC-post COVID		1 (3.3%)				1 (1.8%)
Autoimmune hepatitis	1 (16.6%)					1 (1.8%)
Autoimmune encephalitis				1 (25%)		1 (1.8)%
IDDM				1 (25%)		1 (1.8%)
Lymphoproliferation				1 (25%)		1 (1.8%)
Pyoderma gangrenosum		1 (3.3%)				1 (1.8%)
Langerhans histiocytosis		1 (3.3%)				1 (1.8%)
Alopecia		1 (3.3%)				1 (1.8%)
Cataract		1 (3.3%)				1 (1.8%)
Congenital glaucoma			1 (8.3%)			1 (1.8%)
Total	6 (100%)	30 (100%)	12 (100%)	4 (100%)	3 (100%)	55(100%)

*IBD* inflammatory bowel diseases, *HLH* hemophagocytic lymphohistiocytosis, *AIHA* autoimmune hemolytic anemia, *MISC* multisystem inflammatory syndrome in children, *IDDM* insulin-dependent diabetes mellitus

#### Infections

The reported organisms were all isolated from the patients presenting with infections prior to hospitalization. Regarding total episodes of bacterial infections (*n* = 91), gram-negative bacteria were more frequently isolated than gram-positive bacteria (53.8% and 46.2%, respectively). The most common isolated species were *Staphylococcus* species (37/91 episodes), followed by *Klebsiella* species (18/91 episodes) and *Pseudomonas* species (11/91 episodes) (Table 3).

**Table 3:** Isolated Bacterial species in CGD patients

Organism	No. (91)	Site
Gram-positive bacteria 42/ 91 (46.2%)		
*MRSA*	17	3 (skin*, lung), 2 (ears, blood, LN, liver), 1 (perianal, mediastinal, urine)
*Staphylococcus* species^#^	13	3 skin*, 2 (blood, urine, LN, liver), 1 (lung, bone)
*CONS*	7	5 blood, 1 (mediastinal, CSF)
*Streptococcus pyogenous*	1	Dental abscess
*Alpha hemolytic streptococci*	1	lung
*Enterococcus*	1	Urine
*Anthracoid*	1	Skin*
*Rothia mucilaginosa*	1	Lung
Gram-negative bacteria 49/ 91 (53.8%)		
*Klebsiella* species^#^	10	3 skin*, 2 (lung, blood), 1 (ears, LN, urine)
*Klebsiella pneumonia*	4	2 lung, 1 (blood, perianal)
*Klebsiella MDR*	4	2 urine, 1 (lung, skin*+ bone)
*Pseudomonas aeruginosa*	10	4 lung, 2 (skin*, LN), 1 (urine, blood)
*Pseudomonas MDR*	1	Lung
*Acinetobacter* species^#^	5	3 lung, 2 blood
*Acinetobacter MDR*	2	Skin*, joint
*Acinetobacter baumannii complex*	1	Blood
*Enterobacter*	3	1 lung, 1 LN, 1 skin
*Proteus*	2	1 (ears, skin)
*Stenotrophomonas maltophilia*	1	Skin*
*Serratia*	1	Urine
*Citrobacter*	1	Lung
*Neisseria subflavus*	1	Lung
*Haemophilus influenzae*	1	Lung
*Salmonella species*	1	Blood
*E.coli*	1	Urine

*MDR* multidrug resistance, *MRSA* methicillin resistant Staphylococcus aureus, *CONS* coagulase negative Staphylococcus

#Means the subspecies was not reported

Skin*, skin abscess

One p47^phox^ deficient patient developed at the age of 1 month skin abscess with *Klebsiella* MDR with carbapenem resistance, it was complicated with osteomyelitis which rapidly progressed into gangrene that necessitated amputation. Unfortunately, the patient died from septic shock.

**Fig. 4:**
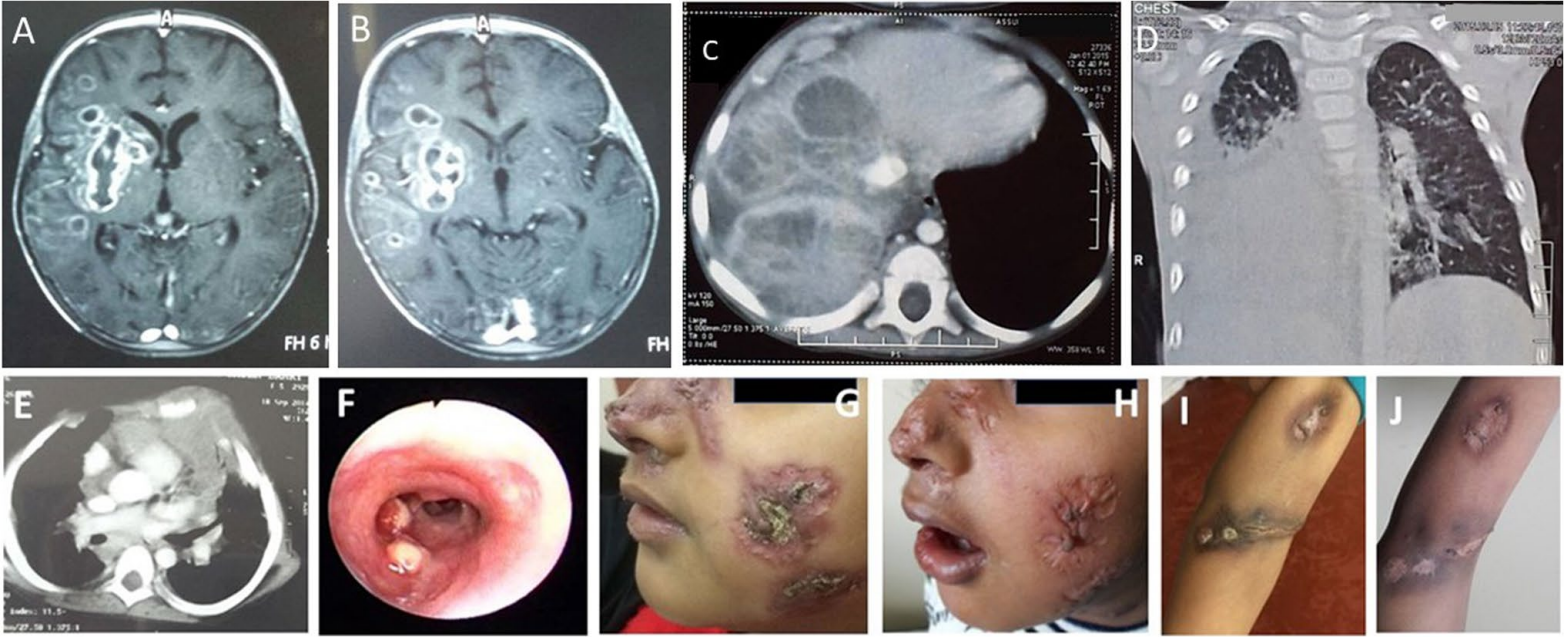
Aspergillus and mycobacterial infections in some patients. A and B, MRI brain T1 post contrast showing right front-temporal marginally enhancing space-occupying lesion; abscess caused by *Aspergillus* infection. C, Post-contrast CT of the abdomen showing right lobe hepatic focal lesion caused by *Aspergillus infection. D*, Shows pulmonary and mediastinal *Aspergillus* infection. E, CT chest showing generalized lymphadenopathy and pulmonary affection due to mycobacterial infection. F, Endoscopy showing bilateral nodular mural lesions in right and left bronchi (atypical mycobacteria). G and I, Mycobacterial skin lesions before treatment. H and J, Mycobacterial skin lesions after anti-tuberculous treatment

*Aspergillus* species isolates were the most common fungal infections in our patients 38/58 (65.5%) followed by *Candida* species 17/58 (29.3%) including *Candida krusei* and *Kodamaea ohmeri* species (Fig. 4) (Table 4).

**Table 4:** Isolated fungal and mycobacterial species in CGD patients

Fungal isolate	No. = 58	Site
*Aspergillus* species^#^	28	16 lung, 3 skin*, 2 brain, 2 bone, 1 mediastinum, 1 liver, 1ear, 1pericardium,1 sinuses
*Aspergillus fumigatus*	8	3 bone, 2 lung, 1 mediastinum, 1 sinus, 1 brain
*Aspergillus flavus*	2	1 sinus, 1 lung
*Candida albicans*	10	5 oral mucosa, 2 lung, 1 tooth, 1 bone, 1 LN
*Candida species*^*#*^	5	2 lung, 1 mediastinum, 1skin, 1 vagina
*Candida dubliniensis*	1	Lung
*Candida krusei*	1	Skin*
*Mucormycosis*	1	Lung
*Fusarium*	1	Lung
*Kodamaea ohmeri*	1	Lung
Mycobacterial isolate	**No. = 44**	**Site**
*Mycobacterium tuberculosis*	24	9 lung, 7 bone, 3 LN, 2 skin*, 2 mediastinum, 1 abdomen
*Bacillus Calmette–Guérin*	13	LN
*Atypical Mycobacteria*	5	2 lung, 1 brain, 1LN, 1 bone
*Mycobacterium immunogenum*	2	lung

*LN* lymph nodes

# Means the subspecies was not reported

Skin*:skin abscess

Regarding the BCG vaccine complication, regional BCG-itis was recorded in 13 patients (7.8%). On the other hand, *Mycobacterium tuberculosis* was the most common isolated *Mycobacterial species* from the infections 24/44 (54.5%) followed by BCG strains 13/44 (29.5%) then *atypical Mycobacterial* species 7/44 (16%). The lung was the most common site of fungal infections, while lymph nodes were the most common site for mycobacterial infections (Fig. 4) (Table 4).

The most common isolated organism from lung infections was *Aspergillus* sp., followed by gram-negative bacteria then *Mycobacterial* species., while the most common organism responsible for skin, subcutaneous and deep organ abscesses (apart from lung and lymph nodes) were gram-positive bacteria, *Mycobacterial* species. followed by *Aspergillus* species., then gram-negative bacteria. On the other hand, BCG strain was the most commonly isolated organism from lymph nodes followed by gram-positive and gram-negative bacteria.

As regards the post-infectious complications, three patients were diagnosed with secondary hemophagocytic lymphohistiocytosis (HLH) at the ages of 3, 5, and 36 months according to the diagnostic criteria for HLH used in the HLH-2004 trial [26]. One of them was secondary to atypical mycobacterial infection, while the other two patients were secondary to bacterial infections. One patient developed multisystem inflammatory syndrome in children (MISC) secondary to COVID-19 infection with coronary affection. Sweet syndrome was diagnosed during the treatment of a 3-month-old P22^phox^ deficient patient with *Klebsiella pneumonia*, *Acinetobacter*, and *Kodamaea ohmeri* infections, and the condition was completely resolved.

#### Treatment

One hundred seventy patients (98.3%) received the prophylactic antibiotics trimethoprim-sulfamethoxazole (TMP-SMX) at a dose of 5 mg/kg/day and Itraconazole as antifungal prophylaxis given at 100 mg once daily (age 5–12 years) or 200 mg/ once daily (age ≥ 13 years). Patients who were younger than 5 years were prescribed Itraconazole after their first fungal infection episode. Three patients did not receive the prophylaxis treatment as they were diagnosed during the acute infection and died. One hundred sixty-nine patients (97.7%) received antimicrobial treatment during the infections. One hundred fifty-seven patients (90.8%) required hospital admission to treat the infections, while 44 patients (25.4%) were admitted to the intensive care unit due to the severity of the infections. Surgical procedures were performed on 85 patients (49.1%) in the form of lymph node biopsy or surgical drainage of the abscess. Thirty-four patients (19.7%) received systemic steroids as a treatment, mostly due to the chest condition. Only 6 patients (3.5%) underwent hematopoietic stem cell transplantation (HSCT).

Regarding the treatment of non-infectious manifestations, patients with IBD received glucocorticoids, mesalamine, and azathioprine as a line of treatment. Patients with non-infectious polyarticular arthritis responded to non-steroidal anti-inflammatory drugs (NSAID) and steroids. The XL-CGD patient who developed IBD, autoimmune hepatitis, and autoimmune hemolytic anemia was treated with steroids and improved. The p67^phox^ deficient patient who developed autoimmune encephalitis received IVIG and steroids and recovered without any neurological sequelae. Two patients who developed secondary HLH received the HLH protocol and improved, while the third patient succumbed to the infections. The patient who developed MISC secondary to COVID-19 infection with coronary affection received IVIG (2 gm/kg), steroids, and acetylsalicylic acid.

#### Outcome

Thirty-six patients (20.8%) were lost to follow-up (18 patients returned to their original countries). Among patients with known outcome, 94/137 patients (68.6%) are living, while the mortality rate was 31.4% (43/137 patients) over a period of 10 years. Pneumonia was the most common cause of death in 32 patients (74.4%), and the causative organisms were the following: 16/32 bacteria, 10/32 invasive aspergillosis, 4/32 *Mycobacterial* species, 1 *Mucormycosis*, and 1 *Candida* sp. resistant to Azole, followed by septicemia in 7 patients (16.2%), secondary HLH in 1 patient (2.3%), failure to engraftment post-HSCT in 1 patient (2.3%), and unknown causes in 2 patients (4.6%).

On comparing the AR and the XL groups, as shown in Table 5, the XL group symptomatized at an earlier age than the AR group (*p* = 0.029). No difference was found between the two groups in the clinical manifestations or the outcome.

**Table 5:** Comparison between AR and X-linked CGD groups

	X-linked (25)	AR (132)	*p*-value
Positive consanguinity	6 (24%)	121 (91.7%)	0.0001
Age of onset (months)/range	3 (0.2–48)	8 (1–180)	0.029
Age of diagnosis (months)/range	24 (1–168)	55 (1–186)	0.12
Diagnosis delay (months)	35 ± 42.5	37.25 ± 39.2	0.8
Abscesses	13 (52%)	74 (56%)	0.4
Pneumonia	19 (76%)	102 (77.3%)	1
Failure to thrive	9 (36%)	67 (50.8%)	0.19
Perianal abscess	4 (16%)	9 (6.8%)	0.13
CNS infections	1 (4%)	17 (12.9%)	0.3
Otitis media	6 (24%)	28 (21.2%)	0.46
Sinusitis	6 (24%)	25 (18.9%)	0.366
Diarrhea	11 (44%)	34 (25.8%)	0.09
Genito-urinary	4 (16%)	5 (3.8%)	0.5
Auto-inflammatory manifestations	4 (16%)	41 (31%)	0.09
Hospital admission	22 (88%)	122 (92.4%)	0.43
ICU admission	6 (24%)	36 (27.2%)	0.8
Surgical procedures	12 (48%)	68 (51.5%)	0.68
Steroids therapy	2 (8%)	32 (24.2%)	0.009
Outcome for followed up patients			
Died	7 (41.2%)	31 (27.7%)	0.075
Living	10 (58.8%)	81 (72.3%)	
Lost from follow-up	8 (36.3%)	20 (15%)	
Overall Survival (years)	6.37 ± 5.29	8.1 ± 4.9	0.2
Age of death (years)	7.25 ± 7.2	4.8 ± 3.7	0.21
Stimulation index of DHR	1.7 (0.73–49)	4.4 (1–33)	0.59

Additionally, patients were subdivided according to defective NADPH components into four groups and compared, as shown in Table 6. p47^phox^ deficient patients exhibited the highest SI of DHR (*p* = 0.007), the highest age at presentation (*p* = 0.001), and overall survival (*p* = 0.001), whereas gp91^phox^ deficient patients showed the lowest SI. It was found that individuals with p22^phox^ and gp91^phox^ defects manifested and were diagnosed earlier than p47^phox^ defective patients.

**Table 6:** Comparison between the four subgroups of CGD according to the deficient component

	Gp91^phox^ (25)	P47^phox^ (83)	P67^phox^ (5)	P22^phox^ (44)	*p*-value
Positive consanguinity	6 (24%)	78 (94%)	5 (100%)	86.4%	0.001
Sex female/male	0/25	40/43	2/3	20/24	0.0001
Age of onset (months)/range	3 (0.2–48)	21 (1–180)	3 (1–60)	2 (1–72)	0.00
Age of diagnosis (months)/range	24 (1–168)	84 (2–186)	48 (2–132)	12 (1–132)	0.00
Diagnosis delay (months)	35 ± 42.5	48 ± 43.5	34.2 ± 27.2	17 ± 18.5	0.0001
Failure to thrive	9 (36%)	42 (50.6%)	2 (40%)	23 (52.2%)	0.55
Perianal abscess	4 (16%)	2 (2.4%)	1 (20%)	6 (13.6%)	0.04
Organomegaly					
Hepatomegaly	2 (8%)	14 (16.8%)	0 (0%)	4 (9%)	0.005
Hepatosplenomegaly	8 (32%)	13 (15.6%)	1 (20%)	19 (43.1%)	
Splenomegaly	1 (4%)	0 (0%)	0(0%)	4 (9%)	
CNS infections	1 (4%)	7 (8.4%)	1 (20%)	9 (20.4%)	0.11
Sinusitis	6 (24%)	17 (20.4%)	0 (0%)	8 (18%)	0.6
Genito-urinary	4 (16%)	4 (4.8%)	1 (20%)	10 (22.7%)	0.024
Inflammatory bowel disease	1 (4%)	3 (3.6%)	0 (0%)	5 (11.4%)	0.29
Diarrhea	11 (44%)	13 (15.6%)	2 (40%)	19 (43%)	0.002
Skin abscess	19 (76%)	53 (63.8%)	3 (60%)	30 (68.2%)	0.7
Sepsis	6 (24%)	14 (16.8%)	1 (20%)	24 (54.5%)	0.00
Lymphadenitis	13 (52%)	40 (48.2%)	0 (0%)	30 (68.2%)	0.01
Osteomyelitis	9 (36%)	20 (24%)	2 (40%)	11 (25%)	0.5
Pneumonia	19 (76%)	68 (81.9%)	4 (80%)	30 (68.2%)	0.37
Regional BCG-itis	3 (12%)	2 (2.4%)	0 (0%)	8 (18%)	0.016
ICU admission	6 (24%)	18 (21.6%)	2 (40%)	16 (36.4%)	0.29
Hospital admission	22 (88%)	76 (91.5%)	5 (100%)	41 (93.1%)	0.79
Surgical procedure	12 (48%)	46 (55.4%)	2 (40%)	20 (45.4%)	0.55
Steroids therapy	2 (8%)	26 (31.3%)	1 (20%)	5 (11.3%)	0.018
Outcome for followed up patients					
Died	7 (41.2%)	14 (18.7%)	1 (20%)	16 (50%)	0.001
Living	10 (58.8%)	61 (81.3%)	4 (80%)	16 (50%)	
Lost from follow-up	8 (36.3%)	8 (9.6%)	0 (0%)	12 (27.3%)	
Overall survival (years)	6.3 ± 5.2	9.75 ± 4.4	7.1 ± 5.1	4.25 ± 4.1	0.001
Age of death (years)	7.25 ± 7.2	6.4 ± 3.59	0 (0%)	3.6 ± 3.5	0.11
Stimulation index of DHR	1.7 (0.73–49)	5.9 (1.19–33)	2.5 (1.4–4.4)	2.7 (1–22.9)	0.007
No. of isolated infectious episodes					Total
Bacterial	19 (21.8%)	41 (47.2%)	1 (1.2%)	26 (29.8%)	87
Fungal	6 (10.9%)	34 (61.8%)	0 (0%)	15 (27.3%)	55
Mycobacterial	3 (7.7%)	23 (59%)	0 (0%)	13 (33.3%)	39

The p22^phox^ and gp91^phox^ deficient patients had the highest percent of gastrointestinal tract (GIT) symptoms (perianal abscess, organomegaly, diarrhea, IBD), and regional BCG-itis with a significant statistical difference, while mycobacterial infections were highest among P47^phox^ deficient followed by P22^phox^ deficient patients (59% and 33.3%, respectively).

Also, we report that the p22^phox^ deficient patients had the highest mortality rate (50%), while the p47^phox^ deficient patients had the lowest (18.7%).

### Prenatal Diagnosis

Genetic counseling was offered to families with known genetic diagnosis; four families sought prenatal diagnosis. Five chorionic villous samples and one amniotic fluid were taken from pregnant mothers in six pregnancies. Maternal contamination was ruled out by variable number tandem repeats (VNTRs) or human leukocyte antigen (HLA) typing for the mother and the fetal samples [24, 25]. Three pregnancies with affected fetuses were terminated due to the unavailability of HLA-matched donors. Three pregnancies were continued with fetuses having the variants in heterozygous forms.

## Discussion

CGD is an inherited primary immunodeficiency disorder of phagocytes; few studies have documented CGD patients from Egypt [15, 16]. Herein, we report 173 patients, which is the largest cohort being reported from Egypt. The significant prevalence of AR forms of CGD (76.3%) is related to the high rate of consanguinity (81.5%), consistent with data from Egypt, Israel, Iran, and Turkey that were previously published [15, 25–29]. The male-to-female ratio in the AR group was 53 to 46% which is similar to the world-wide’s reports [30, 31]. The median age at first manifestation in our cohort was 7 months (ranging from 0.2 to 180 months), which is consistent with studies in India, Mexico, and Israel stating that the first presentations were in the first year of life [10, 28, 32]. While the median age at diagnosis was 48 months, similar to what had been reported in Israel’s and Palestine’s reports [33], it was around 2 years in India and Europe [10, 12] and 30 months in Mexico [32]. Patients with XL-CGD had an earlier age at presentation compared to AR-CGD patients. Patients with p22^phox^ and gp91^phox^ deficiency presented and were diagnosed earlier than p47^phox^ deficient patients. Several reports agreed with our data [10, 33]. However, other countries reported otherwise on comparing between XL and AR groups [30, 34]. The p47^phox^ group had significantly higher SI as well as overall survival rate, and these findings were reported in several previous studies [30, 33]. The plausible explanation is that in the absence of p47^phox^, some electrons are immediately transferred from the FAD prosthetic group in gp91^phox^ to oxygen, directly producing some H_2_O_2_. The small quantities of reactive oxygen species (ROS) appear to be adequate to cope with some infections [35]. There was no difference in either the survival or the mortality rates between the XL and AR groups. Nevertheless, a significant statistical difference was noted in the mortality and survival rates between the four groups. The p47^phox^ deficient patients had the longest overall survival rate, which can be attributed to the same explanation regarding SI. Also, the delay in diagnosis of patients with AR-CGD and lack of awareness among physicians with the mild AR phenotype could explain the missed undiagnosed cases; this contributed to the statistical indifference in the survival and mortality rates between the XL and AR groups. It is important to mention that we diagnosed three male patients with gp91^phox^/ p22^phox^ deficiency, and their mothers were not available to be tested for carrier pattern with the possibility of being CYBC1 deficient patients. It is well known that CYBC1 deficiency can lead to reduced expression of NADPH oxidase’s main subunit (gp91^phox)^ [7].

The most common isolated organism from pulmonary infections was *Aspergillu*s sp., followed by gram-negative bacteria then *Mycobacterial* sp., like in many reports [10, 29, 30]. While we reported that the lungs, followed by the skin, and subcutaneous and deep organ abscesses were the most common sites of fungal infections in our cohort, there was only one lymph node fungal isolate. Another study reported that the lung and lymph nodes were the most common sites [36], and others reported that lymph nodes were the leading infection site [29, 37]. The clinical presentations of fungal infection in CGD were highly mutable, ranging from indolent with minimal symptoms to severe life-threatening infections [38]. Fungal CNS infections could be the first presentation of CGD patients, as we had two patients, one with a brain space-occupying lesion, while the second patient had brain and spinal cord involvement. So, the identification of invasive fungal infection in a patient with unknown risk factors should raise attention for further investigation [9].

Although *Candida krusei* was reported previously as an infectious organism in severe combined immunodeficiency, combined immunodeficiency, and immunocompromised patients [9, 39], to the best of our knowledge, it is the first report of *Candida krusei* in CGD patients. The peculiar importance of this organism is its intrinsic resistance to fluconazole [39]. *Kodamaea ohmeri* which inhabits the environment has emerged during the last decades as a human pathogen that can cause life-threatening infections especially in immunocompromised patients [40, 41]. We reported a 3-month-old P22^phox^ deficient patient who was hospital admitted with pneumonia and liver abscesses. *Kodamaea ohmeri*, *Klebsiella pneumonia,* and *Acinetobacter* were isolated from the bronchoalveolar lavage. During the treatment, the patient developed drug-induced sweet syndrome which was diagnosed according to the diagnostic criteria [42], and the condition resolved completely after treatment. To the best of our knowledge, this is the first report of *Kodamaea ohmeri* infection in primary immunodeficiency diseases.

The wide spectrum of bacterial species that had been isolated from the patients during the infections highlights the importance of proper microorganisms’ isolation and targeted treatment according to sensitivity. *Staphylococcus* species were the most commonly isolated bacteria from the skin and deep organ abscesses followed by *Klebsiella* and *Pseudomonas* species; these infections were similar to other reports [10, 12, 30, 32]. We are raising the alarm about the emergence of the drug-resistant species among the isolated gram-positive and negative bacteria. Drug-resistant bacterial organisms were isolated from 23/91 (25.2%) patients (17 *MRSA*, 4 *Klebsiella MDR*, 1 *Pseudomonas MDR*, and 1 *Acinetobacter MDR*). Antimicrobial resistance is a major global health burden and an urgent problem especially among immunocompromised patients all over the world [43–45].

In addition to infections with the signature microorganisms such as *Aspergillus* and *Staphylococcus* species, *Mycobacterial* species were documented among our patients. In a country with compulsory BCG vaccination in the first month of life, regional BCG-itis was recorded in 13 patients (7.8 %) among the vaccinated patients, which was the first presenting symptom among 4% of them. Similar observation was reported in an Indian report (8%) [10]. While in a Mexican cohort, 58% presented with an adverse reaction, which constituted the first manifestation of CGD in 27 patients (30%), similar to what was reported in Iran [32, 29]. While in Turkey, it was 22% among the vaccinated patients [30], and in Israel, BCG-itis was 2.3% among CGD patients (2 patients from Palestine) as the BCG vaccine is no longer a compulsory vaccine in Israel [28]. The lower percent of BCG complications in our cohort could be attributed to the possibility that minor reaction to BCG vaccine (BCG-itis) induration and ulceration at the site of vaccination, which passed unnoticed or were forgotten by the parents especially in the older age group.

The most common site of mycobacterial infection was the lymph nodes 17/44 (38.6%), followed by the lungs 13/44 (29.5%). Unlike what was reported in the Indian and European experiences as the lung was the most affected site followed by lymph nodes [10, 12]. *Mycobacterium tuberculosis* complex was the most common causative organism 24/44 (54.5%) among the *Mycobacterial* sp. infections followed by BCG then *atypical Mycobacteria*. This was similar to the Indian study [10], while other studies reported that BCG strains were the most common followed by *Mycobacterium tuberculosis* [12, 32, 46]. This difference could be due to the variability in disease endemicity in the different countries. While it was reported in the European experience that there was no association between the occurrence of BCG-itis and CGD type [12], we noticed a statistically significant higher incidence of regional BCG-itis among P22^phox^ deficient patients. It is worth noting that mycobacterial infection was the main presentation among many of the P47^phox^ patients whether with CNS, vertebral, pulmonary, or skin involvement.

In addition to increased susceptibility to infection, patients with CGD have a dysregulated inflammatory response that may require immunosuppressive drugs besides the antimicrobial medications [47, 48]. We reported the hyper-inflammatory clinical manifestations, which included granuloma, IBD, arthritis, secondary HLH, and MISC-post-COVID infection. IBD manifestations were reported in 5.2% of our patients, similar to what was reported in the Indian, Iranian, and Turkish groups [10, 29, 30], but at a lower rate than what was reported in Europe and North America [12, 49]. The difference could be explained by the prevalence of XL-CGD in Western countries, which was associated with a high incidence of colitis [50]. Despite the difficulty in the treatment of IBD in patients with CGD [51], our patients’ responses to treatment were positive.

Genetic diagnosis utilizing Sanger sequencing was done for 44 CGD patients only. It revealed variants in CYBA in 22 patients from 19 different families; almost all of them had the same mutation (p.Val99ProfsTer90), and they originated from the western cities of the Delta and North Coast, while NCF1 mutations were found in 13 patients from 9 different families showing only one variant, p.Tyr26HisfsTer26 in all of them, and they originated from the eastern cities of the Delta. This may point to a founder effect and the importance of the geographic distribution of the patients in correlation with patients’ genotypes.

As Interferon-gamma is not available in Egypt, so it was not used on any of our patients. Despite the antimicrobial prophylaxis, our patients still experienced frequent infections, highlighting the importance of HSCT to CGD patients which was carried out on only 6 patients. The mortality rate in our cohort was 31.4% which is lower than Indian and Mexican reports [10, 32] and higher than those reported in Europe and Israel [12, 28]. Pneumonia was the most common cause of mortality, similar to the European and Indian reports [10, 12]. The overall survival rate in the XL and AR groups of patients is lower than what was reported in Europe, Israel, and Mexico [12, 28, 32]. Nevertheless, the median age of death in both groups was similar to the Mexican report, higher than the Indian report [10], and was lower than what was recorded in Europe and Israel [12, 28].

In conclusion, we documented the different clinical presentations in CGD patients and highlighted the predominance of AR-CGD in our cohort. The delayed diagnosis and the low number of transplanted patients indicate the importance of early diagnosis and initiating proper management for patients to have a better quality of life. The alarm to the emergence of the drug-resistant species is raised, which necessitates antimicrobial stewardship. It is worth mentioning that due to the high incidence of mycobacterial tuberculosis infection among our cohort, any patient with a typical/atypical mycobacterial infection should be screened with a DHR test to role out CGD.

### Study Limitations

The inability to perform DHR testing for all female mothers or to perform intracellular staining for some patients for being lost from follow-up led to the presence of few uncategorized CGD patients especially when molecular diagnosis was not available at the time.

### **Supplementary Information**

ESM 1

## Data Availability

The datasets generated during and/or analyzed during the current study are available from the corresponding author on reasonable request.
