# Supplementary figures and images for: Chronic Granulomatous Disease: a Cohort of 173 Patients—10-Years Single Center Experience from Egypt
Source: J Clin Immunol. 2023 Jul 11;43(8):1799–811. doi: 10.1007/s10875-023-01541-4 (PMC10661789; doi:10.1007/s10875-023-01541-4)

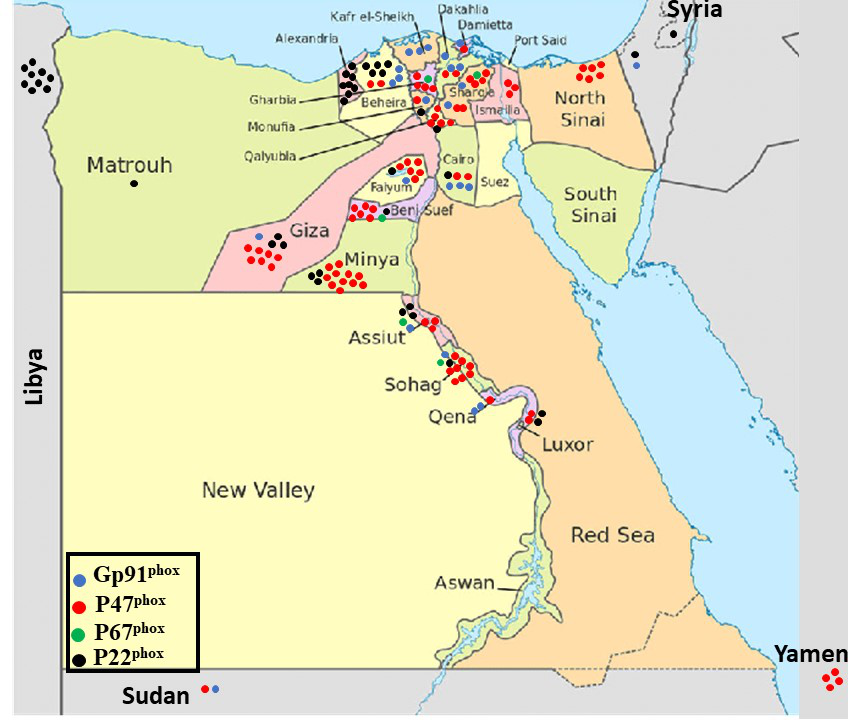

Supplement: Supplementary file 1 — ESM 1 [file 10875_2023_1541_MOESM1_ESM.png]
